# FUNDC1‐Associated Regulation of Mitochondrial Function Is Crucial for Preventing Endothelial Injury in Hyperglycemia

**DOI:** 10.1155/omcl/6619225

**Published:** 2026-06-10

**Authors:** Vinothkumar Rethineswaran, Young Joon Hong, Woong Bi Jang, Jaewoo Choi, Hye Ji Lim, Sangmi Park, Eun Ji Lee, Jong Seong Ha, Jisoo Yun, Sang-Mo Kwon

**Affiliations:** ^1^ Convergence Stem Cell Research Center, Pusan National University, Yangsan, Republic of Korea, pusan.ac.kr; ^2^ Laboratory for Vascular Medicine and Stem Cell Biology, Department of Physiology, School of Medicine, Pusan National University, Yangsan, 50612, Republic of Korea, pusan.ac.kr; ^3^ Department of Cardiology, Chonnam National University School of Medicine, Chonnam National University Hospital, Gwangju, 61469, Republic of Korea, cnuh.com; ^4^ Department of Physiology, College of Medicine, Catholic Kwandong University, Gangneung, Republic of Korea, cku.ac.kr; ^5^ Department of Physiology, School of Medicine, Pusan National University, Yangsan, 50612, Republic of Korea, pusan.ac.kr; ^6^ Research Institute of Convergence Biomedical Science and Technology, Pusan National University Yangsan Hospital, Yangsan, Republic of Korea, pnuyh.or.kr

**Keywords:** ATP synthesis, BAM15, FUNDC1, hyperglycemia, mitochondria

## Abstract

Mitochondria are intracellular organelles that regulate cell survival and death. Hyperglycemia modulates the functioning of the mitochondria in endothelial cells. We discovered that high‐glucose (HG) treatment reduces FUN14 domain‐containing 1 (FUNDC1) expression in endothelial cells. FUNDC1 expression in the mitochondria inhibits the proteasomal degradation of cytochrome C oxidase IV (COX‐IV) and regulates mitochondrial complex I and IV activities as well as ATP synthesis under normal conditions. The FUNDC1 depletion in HG contexts affects mitochondrial complex I and IV activity as well as ATP synthesis and promotes mitochondrial damage through the loss of mitochondrial membrane potential and the production of reactive oxygen species (ROS). BAM15 is a mitochondrial uncoupler that increases mitochondrial function and endothelial survival. Cotreatment with HG and BAM15 increased the FUNDC1 protein expression level and the mitochondrial translocation of FUNDC1 in HG‐treated cells. The BAM15‐induced upregulation of FUNDC1 expression increased the mitochondrial expression of COX‐IV, complex I and IV activity, and ATP synthesis. Our findings suggest that FUNDC1 expression in endothelial cells under hyperglycemic stress plays a crucial role in limiting vascular damage and apoptotic cell death. We discovered a mechanism through which BAM15 protects endothelial cells through FUNDC1‐mediated mitophagy and metabolic regulation. Targeting FUNDC1 via mitochondrial uncoupling is a promising therapeutic strategy for treating diabetic vascular diseases.

## 1. Introduction

Diabetes mellitus (DM) is a chronic metabolic disorder characterized by hyperglycemia and is caused by insulin deficiency or resistance. The vascular endothelium maintains normal vascular tone and blood fluidity under normal conditions; however, the vascular endothelium’s functions are impaired in diabetic conditions [1]. Mitochondrial dysfunction is a precursor of endothelial dysfunction in patients with type 1 and 2 DM. The mitochondrial function and expression of genes encoding key enzymes involved in oxidative metabolism appear to change in those with diabetes. Mitochondria are intracellular organelles that regulate ATP synthesis through glucose and lipid metabolism. Mitochondria play important roles in cell survival and death [2, 3]. Mitochondria generate the majority of cellular energy in the form of ATP via the mitochondrial complex proteins (I, II, III, IV, and V) found in the inner mitochondrial membrane. Mitochondrial complex proteins I, III, and IV are required for producing cellular energy during oxidative phosphorylation. The terminal protein in the electron transport chain, cytochrome C oxidase complex IV, maintains a low and healthy mitochondrial membrane potential in a relaxed state and prevents the formation of mitochondrial reactive oxygen species (ROS) [4, 5]. Impaired cytochrome C oxidase complex IV activity in cells compromises the mitochondrial membrane potential, lowers the ATP levels, and leads to mitochondrial dysfunction [6]. Although mitochondrial complex proteins are linked to the pathogenesis of diabetes, the relationship between diabetes and cytochrome C oxidase IV (COX‐IV) remains poorly understood.

FUN14 domain‐containing 1 (FUNDC1) is a mitophagy receptor protein expressed on the outer mitochondrial membrane. FUNDC1 activates mitophagy through interacting with LC3 via its LC3‐interacting region. FUNDC1 overexpression induces mitophagy in various cell lines, whereas FUNDC1 silencing inhibits hypoxia‐induced mitophagy [7, 8]. FUNDC1‐mediated mitophagy activation prevents myocardial apoptosis during ischemia. FUNDC1 dephosphorylation at Ser13 promotes mitophagy and protects the endothelial cells from damage and apoptosis. FUNDC1 plays an important role in regulating mitochondrial metabolism, and FUNDC1 deficiency reduces mitochondrial quality and worsens diet‐induced obesity. FUNDC1 is essential in endothelial cells for regulating the communication between the endoplasmic reticulum and mitochondria through forming mitochondria‐associated membranes as well as promoting angiogenesis and neoangiogenesis [9]. FUNDC1 knockout inactivated mitophagy in mice fed a high‐fat diet [10–12]. Maintaining mitochondrial homeostasis requires optimal mitophagy. However, the mechanisms through which FUNDC1 regulates mitochondrial complex activity, cellular respiration, and ATP production are unknown.

Mitochondrial coupling involves the use of electron transport to drive ATP synthase to synthesize ATP. Mitochondria are a major source of cellular ROS. Electrons are transferred to the oxygen in the electron transport chain to form water. Electrons leak from the electron transport chain, producing ROS [13, 14]. Mitochondrial uncoupling protects cells against mitochondrial‐damage‐induced ROS generation and cell death [15, 16]. Various conditions cause mitochondrial uncoupling. The uncoupling of mitochondrial respiration inhibits the coupled electron transport and oxidative phosphorylation reactions, which inhibits ATP synthesis without affecting the respiratory chain or ATP synthase. Synthetic mitochondrial uncouplers such as BAM15, CCP, FCCP, 2,4‐dinitrophenol (DNP), and FFA have been used to induce mitochondrial uncoupling in vitro [13]. Obese mice treated with DNP expended more systemic energy due to the decreased proton gradients of the inner mitochondrial membrane, and nutrient oxidation for ATP production was markedly reduced. The clinical application of DNP is limited due to its unsuitable pharmacokinetic properties and side effects [17, 18]. The mitochondrial uncoupler BAM15 highly tolerated previously discovered uncouplers in a laboratory application. BAM15 protects against obesity through increasing energy expenditure, as well as glucose and lipid metabolism, preventing diet‐induced obesity. BAM15 treatment had no off‐target effects on mice with high‐fat‐diet‐induced obesity [19, 20]. BAM15 substantially increased endothelial cell survival in high‐glucose (HG)‐treated cells through increasing the mitochondrial membrane potential and decreasing ROS formation in this study. BAM15 treatment increased FUNDC1 protein expression levels and the mitochondrial translocation of FUNDC1. BAM15 treatment increased mitochondrial function via upregulating FUNDC1 and COX‐IV levels, mitochondrial complex I and IV activities, and ATP synthesis. Overall, our findings suggest that FUNDC1 expression is important for preventing HG‐induced vascular damage.

## 2. Materials and Methods

### 2.1. Cell Culture and Survival

Human umbilical vein endothelial cells were purchased from ATCC and cultured with an EGM‐2 bullet kit system (#CC‐4147, Lonza) containing endothelial basal medium 2 (#CC‐3156, Lonza), 5% fetal bovine serum, human vascular endothelial growth factor, human basic fibroblast growth factor, human epidermal growth factor, human insulin‐like growth factor‐1, and ascorbic acid. FUNDC1‐silenced or ‐overexpressing cells were treated with or without high amounts of glucose. Cell survival was detected using a D‐Plus Cell Counting Kit (CCK‐8) according to the manufacturer instructions (#CCK‐3000, Dongin Biotech). Cells were transfected or treated; the cells were then seeded into 96‐well plates and incubated overnight at 37°C in a 5% CO_2_ incubator. The cells were treated with WST‐1 at 10 μL/well for 1 h in the dark at 37°C in a 5% CO_2_ incubator. The absorbance of each well was measured at 450 nm using a Tecan microplate reader (XFluor, Switzerland). Each experiment was repeated 3–6 times. NG indicates normal glucose, where the normal culture medium was replaced after HG treatment; HG indicates HG, where the culture medium included 25 mM D‐(+) glucose.

### 2.2. Isolation of Crude Mitochondria

The mitochondria were isolated using a previously reported method. In brief, Huvec cells were collected and resuspended in ice‐cold IB cell‐1 buffer (225 mM mannitol, 75 mM sucrose, 0.1 mM EGTA, and 30 mM tris‐HCl, at pH 7.4). Cells were mildly homogenized with a Dounce homogenizer (~200 times). The cell extracts were centrifuged at 600 × *g* for 5 min at 4°C. The supernatant was collected for investigation. The pellet containing unbroken cells and nuclei was discarded. The supernatant was centrifuged again at 600 × *g* for 5 min at 4°C. The supernatant was transferred to a fresh tube and centrifuged at 7000 × *g* for 10 min at 4°C to obtain the crude mitochondrial pellet. The supernatant containing the cytosolic fraction was discarded. The crude mitochondrial pellet was gently resuspended in ice‐cold MRB buffer until the experiments (250 mM mannitol, 5 mM HEPES, and 0.5 mM EGTA at pH 7.4).

### 2.3. Myc‐FUNDC1 Transfection

The endothelial cells were transfected with 1.5 μg/mL of Myc‐FUNDC1 using Megatrans 2.0 transfection reagent according to the manufacturer protocol (ORIGENE). The medium containing the transfection reagent was replaced with fresh medium 3 h after transfection in preparation for further experiments.

### 2.4. siRNA Transfection

The endothelial cells were transfected with FUNDC1 siRNA targeting humans in serum‐reduced Opti‐MEM. The cells were transfected with lipofectamine RNAiMax transfection reagent according to the instructions provided by the manufacturer. The media containing the transfection reagents were removed following 4 h of incubation with the transfection reagent. Fresh media with and without HG were added in preparation for the subsequent experiments.

### 2.5. Tube Formation Assay

Growth‐factor‐reduced Matrigel (#354230; CORNING) was used for the tube formation assay. GFR‐Matrigel was thawed at 4°C 1 day before the experiment, and 96‐well plates were coated with Matrigel at 60 μL/well and polymerized for 30–45 min at 37°C in a CO_2_ incubator. FUNDC1‐overexpressing cells were maintained with or without HG for 48 h. Then, the cells were labeled with 200 nM MitoTracker Green FM (#M7514, Life Technologies) and 200 nM LysoTracker deep red (#12492, Invitrogen) for 30 min at room temperature in the dark. MitoTracker‐ and LysoTracker‐labeled cells were collected and seeded in Matrigel‐coated wells at a density of 6 × 10^3^ cells/well in full medium. The cells were incubated for 6 h at 37°C in a CO_2_ incubator, and then the branch formation was captured using a Lion Heart FX automated microscope (Biotek, USA) at 4 × magnification using different microscopic channels. Branch formation was quantified using ImageJ software.

### 2.6. Annexin V Fluorescein Isothiocyanate (FITC)/Propidium Iodide (PI) Staining

Flow cytometry was performed to quantify mitochondrial apoptosis using an FITC annexin V apoptosis detection kit (#556547, BD Pharmingen). FUNDC1‐overexpressing or ‐silenced cells were collected and seeded onto six‐well plates and incubated with or without the HG treatment for 48 h at 37°C in a CO_2_ incubator. The cells were stained after treatment with annexin V FITC and PI for 15 min at room temperature in the dark and analyzed using fluorescence‐activated cell sorting (FACS) (BD Facs canto II, San Jose, CA, USA). annexin V FITC‐negative and PI‐negative cells were viable, whereas Doublet‐positive cells were apoptotic.

### 2.7. Immunoblotting

Whole‐cell lysates were extracted using Pierce RIPA buffer (#89901, Thermo Fisher Scientific) with a protease and phosphatase inhibitor cocktail. Protein concentrations were determined using a bicinchoninic acid assay kit. The protein samples were separated using SDS‐PAGE (6%–15% gel) and transferred to polyvinylidene difluoride membranes (#IPVH00010, Immobilon‐P‐Millipore). The membranes were blocked with 5% skim milk for 1 h and incubated overnight at 4°C with primary antibodies: FUNDC1 (#49240); BAX (#2772S); cleaved caspase 3 (9661S); LC‐3 A/B (#12741S); phosphor form of AKT, P70S6K, and 4E‐BP1; and total form of AKT, P70S6K, and 4E‐BP1 (all from Cell Signaling technology); FUNDC1 (#bs‐3227R); Bioss. TOMM20 (#sc‐17764); Beta‐Actin (#sc‐47778) (all from Santa Cruz Biotechnology); p62 (#NBP1‐48320); LA‐3B (#NB100‐2220) (both from Novusbio); and DDK (#TA50011) (ORIGENE). The incubation was followed incubation with horseradish‐peroxidase‐conjugated goat anti‐rabbit immunoglobin G (IgG) (#ADI‐SAB‐300‐J) and goat anti‐mouse IgG (#ADI‐SAB‐100‐J) from Enzo Life Sciences. The secondary antibodies at 25°C for 1 h. Western blotting was performed, and the results visualized analyzed using chemiluminescent detection reagent (#WBLUR0500, Immobilon, Millipore).

### 2.8. In Vitro Immunofluorescence Staining

The endothelial cells were grown on poly‐L‐lysine (#P4707; Sigma)‐coated coverslips for immunofluorescence staining. The cells were transfected with FUNDC1 once the cells reached 70%–80% confluence. The transfected cells were either exposed or not to HG levels for 48 h and labeled with 200 nM MitoTracker deep red (#M22426, Invitrogen) for 30–60 min. The cells were then fixed with 4% PFA or 100% cold methanol for 5–10 min at 25°C. The cells were permeabilized with 0.1% Triton X‐100 in 1 × phosphate‐buffered saline (PBS) for 10 min and blocked with 1% bovine serum albumin in 0.3 M glycine for 1 h. The cells were then stained with primary antibodies against LAMP2 (#sc‐18822) and Tom20 (#sc‐17764) from Santa Cruz Biotechnology or LC‐3B (#NB100‐2220) from Novusbio overnight at 4°C in the dark. The cells were then washed three times with PBS (each 5 min in duration) and then incubated with Alexa Fluor‐conjugated secondary antibodies (#A11001) from Invitrogen for 1 h at room temperature in the dark. The cells were then covered with a coverslip using Vectashield vibrance mounting medium (#H‐1700, Vector Laboratories), and images were captured using a Lion Heart FX automated microscope (Biotek, USA) at 40 × magnification.

### 2.9. Reverse‐Transcription Polymerase Chain Reaction (RT‐PCR)

RT‐PCR was performed to examine the effect of BAM15 on the HG‐ and NG‐stimulated cells. The total RNA was purified using TRIzol reagent (Invitrogen, Carlsbad, CA, USA). RNA (1.0 g) was reverse‐transcribed using M‐MLV reverse transcriptase to synthesize complementary DNA. Reverse transcription was performed according to the manufacturer instructions for a PrimeScript 1st strand cDNA synthesis kit (TAKARA). PCR was performed to amplify the complementary DNA using specific primers and the following cycle parameter: denaturation at 94°C for 30 s, annealing at 59°C for 30 s, and extension at 72°C for 30 s. The amplification products after 40 cycles were electrophoresed on 1% agarose gel and visualized using EtBr staining. A UV transilluminator was used to photograph and analyze the gray value of the mRNA expression levels in each group.

### 2.10. Tetramethylrhodamine Ethyl Ester (TMRE) Assay

The mitochondrial membrane polarization was determined using TMRE probe dye. FUNDC1‐silenced or ‐overexpressing cells were exposed to HG treatment and loaded with TMRE probe for 30 min at 37°C in a culture medium. The cells were incubated with the TMRE probe and then washed with PBS. Images of the cells were captured in the RFP channels using a Lion Heart FX automated microscope (Biotek, USA) at 10 × magnification. Mitochondrial depolarization was determined as a decrease in the RFP/GFP fluorescence intensity ratio. Mitochondrial polarization was quantified using Gen5 software with a Lion Heart FX automated microscope (Biotek, USA).

### 2.11. Detection of Cellular and Mitochondrial ROS Levels

The cellular and mitochondrial ROS levels were measured using CellROX Green (#C10444; Invitrogen) and MitoSOX Red reagents, respectively. Briefly, endothelial cells treated with HG levels or BAM15, and the culture media were replaced with fresh media containing 5 μM of CellROX Green reagent or MitoSOX Red reagent and incubated for 15 min at 37°C. Flow cytometry was conducted using FACS (BD FACS Canto 2, San Jose, CA, USA). Fluorescence‐activated cells were sorted using unstained cells as negative controls. The fraction of positively stained cells was determined through comparison with the proportion of unstained cells.

### 2.12. Measurement of Oxygen Consumption Rate (OCR) and Extracellular Acidification Rate (ECAR)

Mitochondrial respiration was evaluated through quantifying the OCR or ECAR using a Seahorse XF extracellular flux analyzer. Briefly, the endothelial cells were treated with HG levels or BAM15. Cells were seeded at 8000 cells/well in cell culture mini plates and incubated at 37°C in a CO_2_ incubator for 1 day before the assay. The assay medium was prepared and supplemented with 1 mM pyruvate, 2 mM glutamine, and 10 mM glucose on the day of the experiment. The OCR and ECAR were determined using 2 µM oligomycin, 4 µM FCCP, and 1 µM rotenone, and 1 µM antimycin A. The OCR and ECAR were measured using a Seahorse XFp analyzer.

### 2.13. Statistical Analyses

Statistical analyses were performed using GraphPad Prism software (Version 5). One‐way analysis of variance (ANOVA) and the paired Student’s *t*‐test were used to assess the differences between the experimental and control groups. Data are presented as the mean ± standard error of the mean (SEM). The results were considered statistically significant at *p* < 0.05 (∗). *p* values less than 0.01 and 0.001 are indicated with ∗∗ and ∗∗∗ in the figures, respectively. The experimental data are presented as the average of three independent experiments.

## 3. Results

### 3.1. HG Promoted FUNDC1 Downregulation in Endothelial Cells

**Figure 1:**
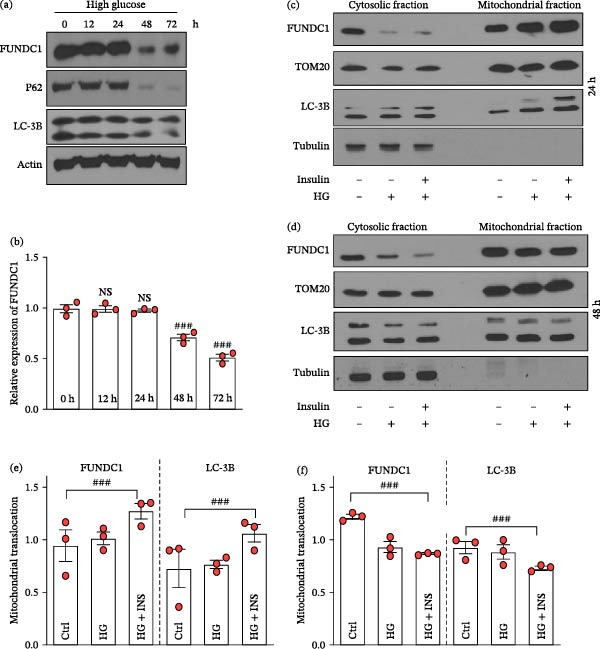
HG treatment downregulates FUNDC1‐mediated mitophagy in endothelial. (a) Immunoblots of FUNDC1, P62, LC‐3B, and actin‐B in endothelial cells cultured under HG conditions for 0, 12, 24, 48, and 72 h as loading controls. (b) Quantification of FUNDC1 protein expression. (c, d) Cells were incubated with HG levels alone or in combination with 1 μM insulin for 24 and 48 h, the cytosolic and mitochondrial fractions were isolated, and western blotting was performed to detect the FUNDC1, TOM20, LC‐3B, and tubulin levels. (e, f) ImageJ software was used to quantify FUNDC1 and LC‐3B protein expression levels. The data were presented as the mean ± standard error of the mean (SEM). At ^#^ *p* < 0.05, the results were considered to be statistically significant. ^##^ *p* < 0.01 or ^###^ *p* < 0.001, with no significant (NS). Each experiment was repeated at least three times.

Endothelial cells were cultured under HG conditions, after which the protein levels of mitochondria, autophagy, and mitophagy‐related markers were measured. The FUNDC1 expression level were lower in the cells maintained under HG conditions compared with those in the NG treatment at several time points (Figure 1a, b), which preceded the inhibition of the ubiquitin marker SQSTM1 and the autophagy marker LC‐3B (Figure 1a). Furthermore, no changes were observed in the protein levels of other mitophagy markers such as PINK1 and BNIP3 after an extended period of HG treatment (Figure S1a). We measured the FUNDC1‐associated mitophagy activation under HG conditions following cotreatment with insulin. Cells treated with HG alone or in combination with insulin showed increased mitochondrial translocation of FUNDC1 and LC‐3B at 24 h compared with that in the basal condition (Figure S2b, c). The cytosol and mitochondria may not have been entirely isolated given the presence of TOM20; however, the crucial observation was the upregulation of the FUNDC1 expression compared with that of the control at 24 h. Long‐term treatment with HG levels alone or in combination with insulin reduced the mitochondrial translocation of FUNDC1 at 48 h compared with that in untreated cells (Figure 1c–f).

### 3.2. FUNDC1 Silencing Promoted Mitochondrial Damage and Apoptosis

**Figure 2:**
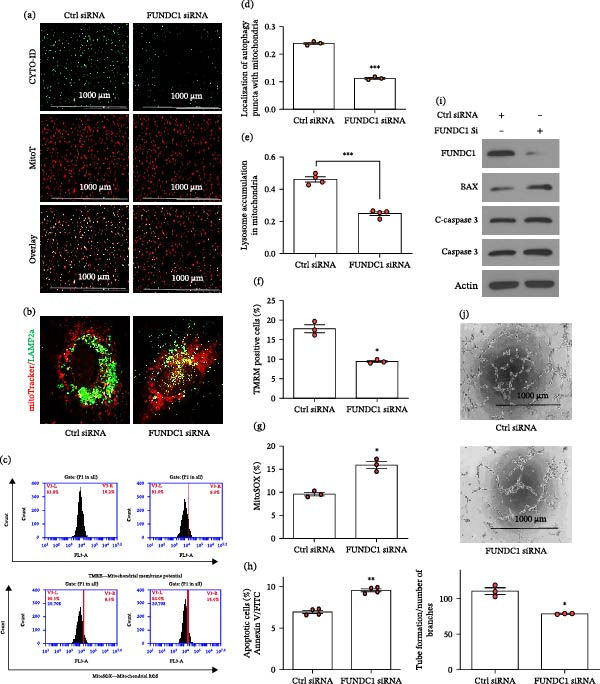
FUNDC1 deletion induced mitochondrial damage, apoptosis, and cell death. (a, b) Representative images and quantification of mitophagy activation using MitoTracker deep red and Cyto‐ID Green colocalization (scale bar, 1000 µm). (c–e) Mitochondrial membrane potential and mitochondrial ROS production were measured. (f) Cells were transfected with FUNDC1 siRNA, and apoptosis was assessed using annexin V FITC/PI staining. (g) Protein levels of mitochondrial apoptosis markers (BAX, caspase 3, and cleaved caspase 3) in FUNDC1‐silenced cells. (h) A vascular network formation assay was performed using the growth‐factor‐reduced Matrigel (scale bar: 1000 µm). ImageJ software was used to quantify vasculature formation in FUNDC1‐silenced and unsilenced cells. The data are presented as the mean ± standard error of the mean (SEM). The results were considered to be statistically significant at ^#^ *p* < 0.05. *p* values less than 0.01 or 0.001 are denoted by ## or ###, respectively, and NS indicates no significant difference. Each experiment was repeated at least thrice. (i) Western blot analysis of FUNDC1 and apoptotic markers. Cells were transfected with Ctrl siRNA or FUNDC1 siRNA, and the expression levels of FUNDC1, BAX, caspase 3, and cleaved caspase 3 (C‐caspase 3) were determined by western blotting. Actin served as a loading control. (j) Representative images and quantification of the vascular tube formation assay. The cells transfected with FUNDC1 siRNA showed a significant reduction in the number of branches compared to the Ctrl siRNA group, as visualized on Matrigel (scale bar: 1000 µm). The bar graph represents the quantitative analysis of tube formation capacity. The data were presented as the mean ± standard error of the mean (SEM). At ^*^ *p* < 0.05, the results were considered to be statistically significant. *p* values less than 0.01 or 0.001 were denoted by  ^∗∗^ or  ^∗∗∗^, with no significance (NS). Each experiment was repeated at least three times.

FUNDC1 is an outer mitochondrial membrane protein. FUNDC1 absence resulted in deficiencies in mitophagy activation, as demonstrated by the colocalization of autophagosome puncta formation determined using MitoTracker (Figure 2a, d, Figure S1b). Our findings showed that the mitochondrial‐lysosome accumulation was notably lower in the siFUNDC1 group than in the control group, as determined via staining and quantification to discern mitochondrial autophagosomes (Figure 2b, e). FUNDC1 silencing also affected mitochondrial membrane potential (Figure 2c, f) and mitochondrial ROS generation (Figure 2c, g), as ROS are byproducts of apoptosis stimulation. We examined whether the FUNDC1‐silencing‐induced reduction in cell survival was caused by apoptosis. Endothelial cell survival was lower and apoptosis levels were higher in FUNDC1‐silencing cells than in the controls (Figure 2h). Furthermore, the levels of apoptosis marker proteins BAX, caspase 3, and cleaved caspase 3 changed (Figure 2i). Furthermore, FUNDC1 silencing consistently reduced vascular branch formation compared with that in the controls (Figure 2j).

### 3.3. FUNDC1 Overexpression Prevented HG‐Induced Mitochondrial Damage, Apoptosis, and Cell Death

Endothelial cells were transfected with Myc‐tagged FUNDC1, and FUNDC1 overexpression was confirmed using western blotting with an anti‐Myc antibody (Figure S2a). FUNDC1 overexpression restored the HG‐induced mitophagy dysfunction. Mitophagy reactivation under HG conditions was observed using MitoTracker, which colocalized with LAMP2 (Figure S2b, c). FUNDC1 overexpression prevented the HG‐induced reduction in mitochondrial membrane potential and the increase in ROS production (Figure S2d–f). FUNDC1 overexpression reduced the percentage of apoptotic cells under HG conditions compared with that in the FUNDC1‐silenced group (Figure S2g, h). FUNDC1 overexpression increased endothelial cell survival in the presence of HG and decreased superoxide dismutase (SOD2) activity (Figure S2i).

### 3.4. BAM15 Regulated Mitochondrial Function and Upregulated FUNDC1 via AKT/mTOR Under HG Conditions

**Figure 3:**
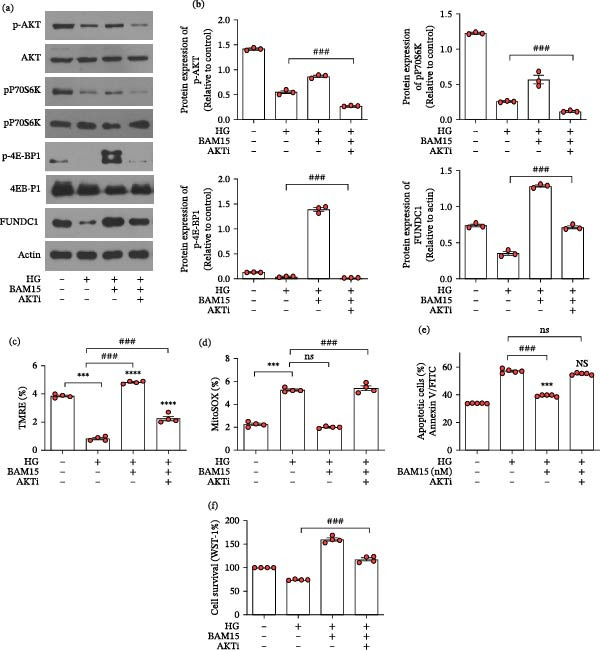
BAM15 AKT‐dependently regulated mitochondrial function in HG‐treated cells. (a) Endothelial cells were stimulated with HG treatment alone or with BAM15 (100 nM) and an AKT inhibitor (0.5 µM) for 48 h. Western blotting was performed to detect FUNDC1 protein expression, phosphorylation, and the total forms of AKT, P70S6K, and 4E‐BP1. (b) Western blot results. (c, d) Evaluation of mitochondrial membrane potential and ROS levels using TMRE and MitoSOX assays. (e, f) Cells were treated with HG levels alone or in combination with BAM15 and an AKT inhibitor (0.5 µM) for 48 h. Cell survival and apoptosis were assessed using annexin V FITC/PI staining and the WST‐1 assay. The data are presented as the mean ± standard error of the mean (SEM). The data were presented as the mean ± standard error of the mean (SEM). Statistical significance was considered at *p* < 0.05.  ^∗^ *p* < 0.05,  ^∗∗^ *p* < 0.01,  ^∗∗∗^ *p* < 0.001, and  ^∗∗∗∗^ *p* < 0.0001 vs. the control group. ^#^ *p* < 0.05, ^##^ *p* < 0.01, and ^###^ *p* < 0.001 vs. the HG‐treated group. “ns” or “NS” indicates no significance. Each experiment was repeated at least three times.

We investigated whether the BAM15‐induced increase in the FUNDC1 expression level was regulated at the mRNA or protein level. We found no differences in the mRNA expression levels of FUNDC1 in cells cotreated with HG and BAM15 at different time points (Figure S3). We then examined whether the BAM15‐induced upregulation of FUNDC1 was dependent on AKT/mTOR. Cells were exposed to HG treatment alone, HG levels in combination with BAM15, or BAM15 plus an AKT inhibitor. We found that the expressions of phosphorylated forms of AKT (Ser473), P70S6K, 4E‐BP1, and FUNDC1 were downregulated in response to HG treatment compared with the baseline. However, HG and BAM15 cotreatment increased the levels of the phosphorylated forms of AKT, P70S6K, 4E‐BP1, and FUNDC1 compared with the pretreatment levels; the total level of phosphorylated forms did not significantly change (Figure 3a, b). The BAM15‐induced increase in the phosphorylation of AKT, P70S6K, 4E‐BP1, and FUNDC1 was suppressed by treatment with an AKT inhibitor (Figure 3a, b). The increased BAM15‐induced mitochondrial function under HG conditions was reversed by treatment with the AKT inhibitor, as evidenced by a decrease in the mitochondrial membrane potential and increases in ROS production as well as apoptosis (Figure 3c–e), (Figure S4a, b). In addition, BAM15 treatment increased cell survival rates under HG conditions (Figure 3f). The expression of the apoptosis marker proteins BAX and cleaved caspase 3 was not increased in the HG‐treated cells compared with those in the NG cells, but when the cells received HG and BAM15 cotreatment, the expression levels of BAX and cleaved caspase 3 were lower than those in the HG‐alone‐treated cells. Treatment with the AKT inhibitor reversed the BAM15‐induced inhibition of apoptosis marker protein expression in the HG‐treated cells (data not shown).

### 3.5. BAM15 Increased Mitochondrial Function via Regulating Cellular Respiratory Kinetics in HG‐Treated Cells

**Figure 4:**
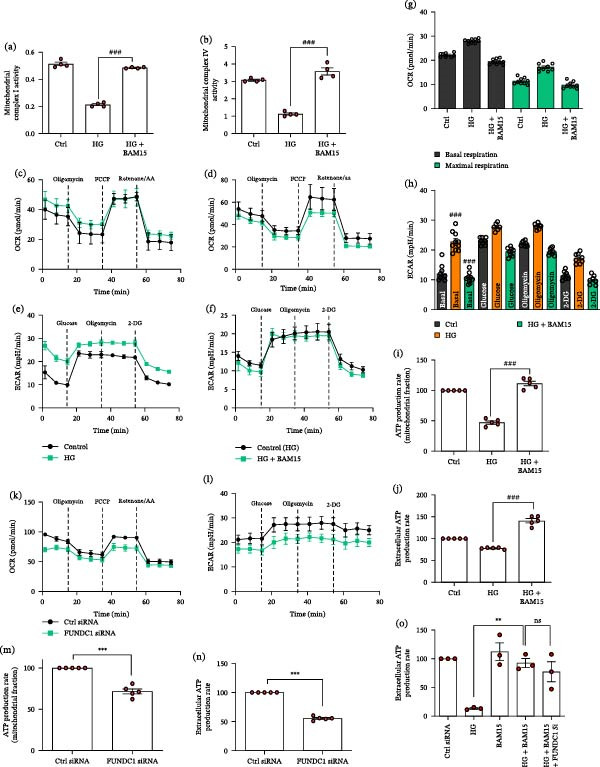
BAM15 preserves mitochondrial efficiency and ATP synthesis under HG stress. (a, b) Activities of mitochondrial complexes I and IV in endothelial cells treated with HG levels and BAM15 for 48 h. (c–h) Measurement of OCR and ECAR using the Seahorse XFp analyzer. Mitochondrial respiration (basal/maximal respiration and ATP‐linked OCR) and glycolytic function were determined via Mito and glycolysis stress tests. (i, j) Quantitative detection of ATP in isolated mitochondria. Briefly, mitochondria were isolated from cells incubated with HG levels and BAM15 for 48 h, and ATP was quantified using an Enliten ATP assay system, as directed by the manufacturer. The amount of ATP released was measured using a luciferase‐based Enliten ATP assay system according to the manufacturer instructions. The data are presented as the mean ± standard error of the mean (SEM). The results were considered to be statistically significant at ^#^ *p* < 0.05. *p* values less than 0.01 or 0.001 are denoted by ## or ###, respectively, and NS indicates no significant difference. Each experiment was repeated at least thrice. (k, l) Measurement of OCR and ECAR in FUNDC1‐silenced cells. Endothelial cells were transfected with Ctrl siRNA or FUNDC1 siRNA, and mitochondrial respiration (OCR) and glycolytic function (ECAR) were monitored using the Seahorse XFp analyzer. (m, n) Quantitative analysis of mitochondrial ATP production rate and extracellular ATP production rate in FUNDC1‐silenced cells. FUNDC1 knockdown significantly reduced both mitochondrial and extracellular ATP levels compared to the control group. (o) Assessment of the synergistic or dependent effects of BAM15 and FUNDC1 on extracellular ATP production. Cells were treated with HG, BAM15, and FUNDC1 siRNA in various combinations to determine the role of FUNDC1 in BAM15‐mediated metabolic recovery under HG stress. The data are presented as the mean ± SEM. Statistical significance is indicated by  ^∗∗^ *p* < 0.01 or  ^∗∗∗^ *p* < 0.001, and NS denotes no significant difference.

The OCR and ECAR were determined using a Seahorse XFp analyzer to determine healthy mitochondrial function. The OCR is an indicator of mitochondrial function (aerobic glycolysis and oxidative phosphorylation), whereas the ECAR is an indicator of cellular glycolytic activity (anaerobic glycolysis). The OCR and ECAR of the HG‐cultured endothelial cells were markedly different (Figure 4a–d). The cells exposed to HG conditions or those cotreated with HG levels and BAM15 showed that their mitochondrial function was altered by modulating the maximal respiratory capacity, spare capacity, and nonmitochondrial respiration (Figure 4e, f). The BAM15 treatment normalized the mitochondrial and ATP‐linked respiration in the HG‐treated cells (Figure 4g). In addition, we measured the ECAR in the cells treated with HG levels alone or in combination with BAM15 to determine the metabolic shift from oxidative phosphorylation to glycolysis. HG treatment increased the ECAR under basal conditions in the absence of BAM15 compared with that in the control cells (Figure 4h, i). We validated the effect of BAM15 treatment on FUNDC1‐mediated mitophagy by conducting another experiment using the siFUNDC1 group. Our findings revealed that ATP production was lower in the group with reduced FUNDC1 expression compared with the control group, mirroring the pattern observed in response to HG levels (Figure 4k–o).

### 3.6. BAM15 Regulated Mitochondrial Function via Regulating Mitochondrial Complex Activity and ATP Synthesis in HG‐Treated Cells

**Figure 5:**
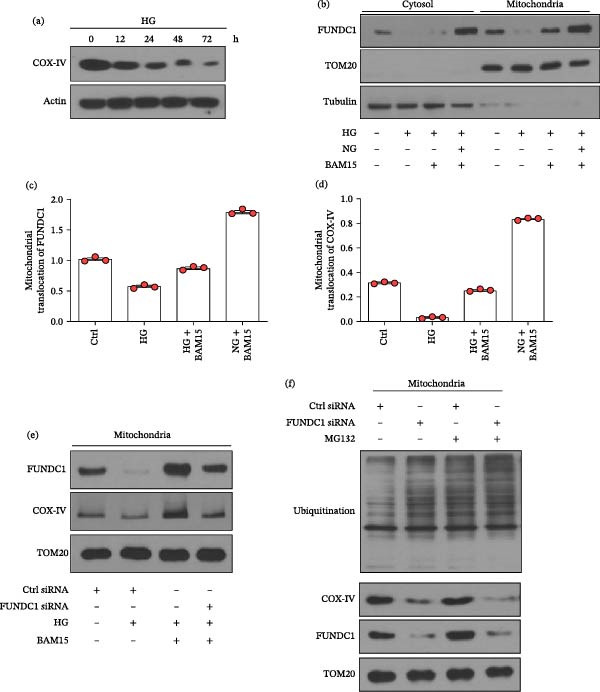
BAM15 promotes COX‐IV stability and FUNDC1 translocation in HG‐treated cells. (a) Cells were treated with high amount of glucose at various time points, and COX‐IV protein expression level was detected using western blotting. (b–d) Cells were cultured for 48 h in NG and HG groups, with or without BAM15 treatment. The cytosolic and mitochondrial fractions were then extracted, and western blotting was performed to detect the cytosolic‐to‐mitochondrial translocation of FUNDC1, LC‐3B, and COX‐IV. (e) BAM15‐induced upregulation of FUNDC1 and COX‐IV was inhibited by FUNDC1 silencing under HG conditions. (f) Cells were treated with HG, FUNDC1 siRNA, and proteasome inhibitor, and western blotting was performed to detect the ubiquitination of FUNDC1, COX‐IV, and TOM20.

The changes in OCR and ECAR under HG conditions compared with those of the control indicated mitochondrial dysfunction. The increased OCR and ECAR indicated an increase in energy demand, which was supported by the decreased activities of mitochondrial complexes I and IV as well as the decreased ATP synthesis in HG‐treated cells compared with those in the control. BAM15 treatment restored mitochondrial function via regulating the OCR and ECAR under basal conditions. Furthermore, BAM15 treatment increased mitochondrial function through enhancing the activity of complexes I and IV as well as increasing ATP synthesis under HG conditions (Figure 4a–h). Furthermore, we quantified the ATP production rate in isolated mitochondria and cell culture media to confirm how the endothelial cells met their energy demands under HG stress. HG treatment reduced the ATP production rate in the isolated mitochondria and conditioned media compared with that in the controls, whereas BAM15 and HG cotreatment increased the ATP production rate in isolated mitochondria and conditioned media (Figure 4i, j). We explored how the ATP production rate was suppressed under HG conditions by measuring the COX‐IV expression levels, which is a critical marker of mitochondrial membrane potential, mitochondrial complex activity, and ATP synthase activity. The COX‐IV expression was lower in the HG‐treated cells at different time points than that in the control (Figure 5a). BAM15 treatment of HG cells induced the regulation and mitochondrial translocation of FUNDC1 and COX‐IV (Figure 5b–d). We determined whether FUNDC1 plays a role in regulating COX‐IV expression in the mitochondria by silencing FUNDC1 with siRNA. Silencing FUNDC1 affected the COX‐IV expression level in the mitochondria under HG and BAM15 cotreatment compared with that in the control (Figure 5e). Next, we explored whether the molecular mechanism through which COX‐IV was reduced in response to HG treatment was proteasome‐dependent. We found that the loss of FUNDC1 expression in the mitochondria reduced the stability of COX‐IV expression in the mitochondria via ubiquitination and subsequent degradation under HG conditions (Figure 5f). FUNDC1 is a major regulator of COX‐IV expression in the mitochondria, regulating mitochondrial function and ATP synthesis under HG conditions.

## 4. Discussion

Mitochondria are metabolic mediators and the major sources of energy for endothelial cells [21]. Hyperglycemia damages the mitochondria; damaged or dysfunctional mitochondria are selectively eliminated via mitophagy, a key component of the mitochondrial quality control system [22–26]. Removing damaged mitochondria is a fundamental process in the system controlling mitochondrial quality, and several mitophagy marker proteins are used to maintain healthy mitochondria via mitochondrial quality control mechanisms [10, 27, 28]. While the PINK1/Parkin and BNIP3 pathways are well‐characterized mediators of mitophagy in various contexts, our study primarily observed their stable expression under certain stress conditions. We found that HG stress downregulated the expression level of the mitophagy receptor FUNDC1 in endothelial cells compared with that in normal cells, leading to a mitochondrial dysfunction cascade characterized by suppressed mitochondrial complex activity and ATP production. These findings are consistent with those of recent studies emphasizing the role of impaired mitochondrial quality control, including mitophagy, in the pathogenesis of diabetic‐related vascular complications [29, 30].

FUNDC1 not only facilitates mitophagy but also participates in angiogenesis and vascular remodeling under pathological conditions [9]. FUNDC1 knockout increases mitochondrial dysfunction as well as insulin resistance and worsens diet‐induced obesity [11]. Moreover, FUNDC1 has been implicated in calcium homeostasis in cardiomyocytes, and its dysregulation may result in cardiac dysfunction [31]. In our study, HG conditions selectively reduced FUNDC1 expression, whereas the expression levels of PINK1 and BNIP3L remained unchanged compared with those in the control. Due to the selective downregulation of FUNDC1 and the lack of significant alterations in the canonical PINK1/Parkin‐dependent pathway in response to HG, we focused our investigation on FUNDC1 as the primary driver of mitochondrial restoration in this model. FUNDC1 silencing further exacerbated mitochondrial damage, lowered the membrane potential, increased the ROS production, and increased apoptotic cell death in HG conditions. Mitochondria are major producers of ROS, which provide signaling functions at physiological levels but cause oxidative damage under HG conditions [13]. Mitochondrial uncoupling is a strategy to mitigate ROS production and improve metabolic profiles. The use of conventional uncouplers, such as FCCP and DNP, is limited by their off‐target effects, such as plasma membrane depolarization. These limitations prompted interest in next‐generation mitochondrial therapeutics, such as metformin, SHC517, MitoQ, urolithin A, and sappanone A [13, 32–37]. These agents affect mitochondrial dynamics, mitophagy, and the antioxidant defense system, ensuring reinforced mitochondrial quality control and energy homeostasis. BAM15 is a promising mitochondrial uncoupler that does not depolarize the plasma membrane and is capable of increasing mitochondrial efficiency and glycemic control [19, 20]. In our study, the low‐dose BAM15 treatment more effectively alleviated the HG‐induced stress in endothelial cells than insulin treatment, increasing survival rates and preserving mitochondrial function. BAM15 treatment of the HG‐treated cells restored their FUNDC1 expression level and promoted FUNDC1 translocation to mitochondria, suggesting that mitochondrial uncoupling enhances mitophagy via the FUNDC1 pathway. This mild uncoupling paradoxically preserves mitochondrial efficiency under stress by preventing mitochondrial hyperpolarization, which is a major source of excessive ROS. By reducing the ROS‐driven damage to the electron transport chain and promoting the selective clearance of dysfunctional mitochondria through FUNDC1, BAM15 maintains a high‐quality mitochondrial pool. Consequently, the remaining healthy mitochondria can operate more effectively, leading to the observed recovery of mitochondrial complex activity and ATP synthesis despite the uncoupling properties of BAM15. This process is distinct from that of traditional uncouplers such as FCCP and CCCP, which primarily activate mitophagy through PINK1/PARKIN signaling [38].

Our data support the hypothesis that BAM15 enhances endothelial function through AKT/mTOR signaling. AKT activation regulates angiogenesis and protein turnover in endothelial cells [39, 40]. BAM15 treatment increased AKT phosphorylation and the levels of downstream effectors, such as p70S6K and 4E‐BP1, indicating a role of the AKT/mTOR axis in mediating FUNDC1 expression and mitochondrial restoration. Whether the activation of FUNDC1 by BAM15 is entirely AKT‐dependent warrants further investigation; however, our findings underscore a link between BAM15, AKT signaling, and mitochondrial repair. BAM15 treatment increased mitochondrial respiration and proton leakage, thereby sustaining ATP levels under stress, in agreement with the findings of prior studies [19, 20]. Although HG stress inhibited the basal and maximal respiration levels, BAM15 treatment reversed these effects and restored cellular energy homeostasis, likely through a compensatory glycolytic shift. COX‐IV is a component of complex IV that is critical for electron transport and membrane potential. The COX‐IV levels decreased by HG stress were also restored by BAM15 treatment. This suggested that mild uncoupling paradoxically enhanced mitochondrial output through metabolic adaptation, possibly supported by alternative substrate use or glycolysis‐derived intermediates [13, 41]. Mild mitochondrial uncoupling can affect mitochondrial function and reduce ATP production. However, our findings showed that mild mitochondrial uncoupling under BAM15 treatment increased the activities of mitochondrial complexes I and IV, as well as the ATP synthesis rate in HG‐treated cells, most likely due to a glycolytic shift that supported mitochondrial complex activity and ATP synthesis. Furthermore, whether the BAM15‐induced upregulation of mitochondrial complex activity and ATP synthesis induced by HG stress is primarily due to a glycolytic metabolic shift or whether other mitochondrial metabolites play a compensatory role in endothelial cells remains unknown and thus warrants further investigation.

In conclusion, we identified BAM15 as a potent modulator of endothelial mitochondrial homeostasis. BAM15 increases endothelial survival and function under HG conditions through upregulating FUNDC1‐mediated mitophagy and activating AKT/mTOR signaling. These findings support BAM15 as a therapeutic candidate for diabetic vascular injury, particularly by addressing mitochondrial dysfunction, which is an unmet clinical need. Nevertheless, this study did not utilize autophagic flux inhibitors to further validate the dynamics of mitophagy, which remains a focus for future research. However, this study was limited to in vitro endothelial cell models; as such, further validation is required using in vivo systems to determine the pharmacokinetics, systemic safety, and therapeutic feasibility of using BAM15 in complex physiological environments. Future studies should also explore the effects of BAM15 treatment in patient‐derived endothelial cells and diabetic organoid models to more accurately predict its translational potential.

## Author Contributions

Vinothkumar Rethineswaran designed the study and established the experimental protocols. Young Joon Hong, Woong Bi Jang, Jaewoo Choi, Hye Ji Lim, Sangmi Park, Eun Ji Lee, Jong Seong Ha, and Jisoo Yun prepared the figures, assisted with data presentation, and contributed to the statistical analyses. Vinothkumar Rethineswaran and Young Joon Hong wrote the manuscript. Sang‐Mo Kwon supervised the project.

## Funding

This study was supported by the National Research Foundation of Korea (NRF) grants funded by the Korean government (MSIT) (Grants RS‐2022‐NR070846 and RS‐2025‐00555693).

## Disclosure

A preprinted version of this manuscript was previously published by Research Square (DOI: 10.21203/rs.3.rs‐2903880/v1) [42].

## Conflicts of Interest

The authors declare no conflicts of interest.

## Supporting Information

Additional supporting information can be found online in the Supporting Information section.

## Supporting information


**Supporting Information** Figure S1: Mitophagy marker expression levels in endothelial cells. (a) Immunoblots of mitophagy markers PINK1, BNIP3, and actin, which were used as loading controls from cells cultured in HG conditions for 24 and 48 h. (b) Proteins were extracted from endothelial cells transfected with siRNA FUNDC1 and control siRNA after 24 and 48 h. The changes in FUNDC1 and actin protein expression levels were visualized using western blotting. The data are presented as the mean ± standard error of the mean (SEM). The results were considered statistically significant at ^#^ *p* < 0.05. *p* values less than 0.01 or 0.001 are denoted with ## or ###, respectively; NS indicates the difference was not significant. Each experiment was repeated at least three times. Figure S2: FUNDC1 overexpression prevented HG‐induced mitochondrial damage, apoptosis, and cell death. (a) Endothelial cells were transfected with Myc‐tagged FUNDC1, and FUNDC1 overexpression was detected using western blotting with anti‐Myc and FUNDC1 antibodies. (b, c) Cells were transfected with Myc‐tagged FUNDC1, and immunofluorescence was used to observe the expression levels of FUNDC1 Myc with LAMP2. (d–f) Mitochondrial membrane potential and mitochondrial ROS levels were measured using a flow cytometer, TMRE assay, and MitoSOX reagents. (g) The amount of apoptosis and numbers of dead cells were measured using annexin V FITC/PI staining. (h) SOD2 activity was measured in FUNDC1‐overexpressing HG‐treated cells. (i) Data are presented as mean ± standard error of the mean (SEM). The results were considered statistically significant at *p* < 0.05 (#). *p* values less than 0.01 or 0.001 are denoted by ## or ###, respectively; NS indicates the difference was not significant. Each experiment was repeated at least three times. Figure S3: FUNDC1 mRNA expression in BAM15‐ and HG‐treated cells. (a, b) Total RNA was isolated and reverse‐transcription PCR was performed to determine the expression levels of FUNDC1 in HG‐ and BAM15‐stimulated cells. FUNDC1 mRNA expression level was quantified and normalized with GAPDH. Cells were cultured for 24 or 48 h under HG conditions, with and without BAM15 and insulin. The RNA was then isolated, and PCR was used to examine the expression levels of FUNDC1 and GAPDH. FUNDC1 mRNA expression level was quantified and normalized using GAPDH. The data are presented as the mean ± standard error of the mean (SEM). The results were considered statistically significant at *p* < 0.05 (#). *p* values less than 0.01 or 0.001 are denoted by ## or ###, respectively; NS indicates the difference was not significant. Each experiment was repeated at least three times. Figure S4: BAM15 treatment increased mitochondrial function via regulating the mitochondrial membrane potential and ROS release in HG‐treated cells. (a) BAM15 treatment regulated mitochondrial function via modulating mitochondrial membrane potential and ROS release in HG‐treated cells, as measured using flow cytometry with TMRE assay, MitoSOX, and CellROX dyes. (b) Apoptosis was assessed in HG‐treated cells treated with or without BAM15 using annexin V FITC/PI staining on a flow cytometer.

## Data Availability

The data that support the findings of this study are available from the corresponding author upon reasonable request.

## References

[1] Rask-Madsen C. and King G. L. , Vascular Complications of Diabetes: Mechanisms of Injury and Protective Factors, Cell Metabolism. (2013) 17, no. 1, 20–33, 10.1016/j.cmet.2012.11.012. 23312281 PMC3546345

[2] Bhatti J. S. , Bhatti G. K. , and Reddy P. H. , Mitochondrial Dysfunction and Oxidative Stress in Metabolic Disorders-A Step towards Mitochondria Based Therapeutic Strategies, Biochimica et Biophysica Acta (BBA)-Molecular Basis of Disease. (2017) 1863, no. 5, 1066–1077, 10.1016/j.bbadis.2016.11.010. 27836629 PMC5423868

[3] Spinelli J. B. and Haigis M. C. , The Multifaceted Contributions of Mitochondria to Cellular Metabolism, Nature Cell Biology. (2018) 20, no. 7, 745–754, 10.1038/s41556-018-0124-1. 29950572 PMC6541229

[4] Martínez-Reyes I. and Chandel N. S. , Mitochondrial TCA Cycle Metabolites Control Physiology and Disease, Nature Communications. (2020) 11, no. 1, 10.1038/s41467-019-13668-3. PMC6941980 31900386

[5] Kluge M. A. , Fetterman J. L. , and Vita J. A. , Mitochondria and Endothelial Function, Circulation Research. (2013) 112, no. 8, 1171–1188, 10.1161/CIRCRESAHA.111.300233. 23580773 PMC3700369

[6] Kogot-Levin A. , Saada A. , and Leibowitz G. , et al.Upregulation of Mitochondrial Content in Cytochrome c Oxidase Deficient Fibroblasts, PLoS One. (2016) 11, no. 10, 10.1371/journal.pone.0165417, e0165417. 27780242 PMC5079646

[7] Zhang W. , The Mitophagy Receptor FUN14 Domain-Containing 1 (FUNDC1): A Promising Biomarker and Potential Therapeutic Target of Human Diseases, Genes & Diseases. (2021) 8, no. 5, 640–654, 10.1016/j.gendis.2020.08.011. 34291135 PMC8278526

[8] Chen Z. , Liu L. , and Cheng Q. , et al.Mitochondrial E3 Ligase MARCH5 Regulates FUNDC1 to Fine-Tune Hypoxic Mitophagy, EMBO Reports. (2017) 18, no. 3, 495–509, 10.15252/embr.201643309. 28104734 PMC5331199

[9] Wang C. , Dai X. , and Wu S. , et al.FUNDC1-Dependent Mitochondria-Associated Endoplasmic Reticulum Membranes are Involved in Angiogenesis and Neoangiogenesis, Nature Communications. (2021) 12, no. 1, 10.1038/s41467-021-22771-3, 2616. PMC8110587 33972548

[10] Pei Z. , Liu Y. , and Liu S. , et al.FUNDC1 Insufficiency Sensitizes High Fat Diet Intake-Induced Cardiac Remodeling and Contractile Anomaly Through ACSL4-Mediated Ferroptosis, Metabolism-Clinical and Experimental. (2021) 122, 10.1016/j.metabol.2021.154840, 154840. 34331963

[11] Wu H. , Wang Y. , and Li W. , et al.Deficiency of Mitophagy Receptor FUNDC1 Impairs Mitochondrial Quality and Aggravates Dietary-Induced Obesity and Metabolic Syndrome, Autophagy. (2019) 15, no. 11, 1882–1898, 10.1080/15548627.2019.1596482. 30898010 PMC6844496

[12] Fu T. , Xu Z. , and Liu L. , et al.Mitophagy Directs Muscle-Adipose Crosstalk to Alleviate Dietary Obesity, Cell Reports. (2018) 23, no. 5, 1357–1372, 10.1016/j.celrep.2018.03.127. 29719250

[13] Demine S. , Renard P. , and Arnould T. , Mitochondrial Uncoupling: A Key Controller of Biological Processes in Physiology and Diseases, Cells. (2019) 8, no. 8, 10.3390/cells8080795. PMC6721602 31366145

[14] Mookerjee S. A. , Divakaruni A. S. , Jastroch M. , and Brand M. D. , Mitochondrial Uncoupling and Lifespan, Mechanisms of Ageing and Development. (2010) 131, no. 7-8, 463–472, 10.1016/j.mad.2010.03.010. 20363244 PMC2924931

[15] Rai Y. , Anita , and Kumari N. , et al.Mild Mitochondrial Uncoupling Protects From Ionizing Radiation Induced Cell Death by Attenuating Oxidative Stress and Mitochondrial Damage, Biochimica et Biophysica Acta (BBA)-Bioenergetics. (2021) 1862, no. 1, 10.1016/j.bbabio.2020.148325, 148325. 33065098

[16] Cadenas S. , Mitochondrial Uncoupling, ROS Generation and Cardioprotection, Biochimica et Biophysica Acta (BBA)-Bioenergetics. (2018) 1859, no. 9, 940–950, 10.1016/j.bbabio.2018.05.019. 29859845

[17] Grundlingh J. , Dargan P. I. , El-Zanfaly M. , and Wood D. M. , 2,4-Dinitrophenol (DNP): A Weight Loss Agent With Significant Acute Toxicity and Risk of Death, Journal of Medical Toxicology. (2011) 7, no. 3, 205–212, 10.1007/s13181-011-0162-6. 21739343 PMC3550200

[18] Goldgof M. , Xiao C. , Chanturiya T. , Jou W. , Gavrilova O. , and Reitman M. L. , The Chemical Uncoupler 2,4-Dinitrophenol (DNP) Protects Against Diet-Induced Obesity and Improves Energy Homeostasis in Mice at Thermoneutrality, Journal of Biological Chemistry. (2014) 289, no. 28, 19341–19350, 10.1074/jbc.M114.568204. 24872412 PMC4094046

[19] Alexopoulos S. J. , Chen S.-Y. , and Brandon A. E. , et al.Mitochondrial Uncoupler BAM15 Reverses Diet-Induced Obesity and Insulin Resistance in Mice, Nature Communications. (2020) 11, no. 1, 10.1038/s41467-020-16298-2, 2397. PMC7224297 32409697

[20] Axelrod C. L. , King W. T. , and Davuluri G. , et al.BAM15-Mediated Mitochondrial Uncoupling Protects Against Obesity and Improves Glycemic Control, EMBO Molecular Medicine. (2020) 12, no. 7, 10.15252/emmm.202012088, e12088. 32519812 PMC7338798

[21] Caja S. and Enríquez J. A. , Mitochondria in Endothelial Cells: Sensors and Integrators of Environmental Cues, Redox Biology. (2017) 12, 821–827, 10.1016/j.redox.2017.04.021. 28448943 PMC5406579

[22] Shan Z. , Fa W. H. , Tian C. R. , Yuan C. S. , and Jie N. , Mitophagy and Mitochondrial Dynamics in Type 2 Diabetes Mellitus Treatment, Aging. (2022) 14, no. 6, 2902–2919, 10.18632/aging.203969. 35332108 PMC9004550

[23] Tong M. , Saito T. , and Zhai P. , et al.Mitophagy is Essential for Maintaining Cardiac Function During High Fat Diet-Induced Diabetic Cardiomyopathy, Circulation Research. (2019) 124, no. 9, 1360–1371, 10.1161/CIRCRESAHA.118.314607. 30786833 PMC6483841

[24] Yang Y. , Li T. , Li Z. , Liu N. , Yan Y. , and Liu B. , Role of Mitophagy in Cardiovascular Disease, Aging and Disease. (2020) 11, no. 2, 419–437, 10.14336/AD.2019.0518. 32257551 PMC7069452

[25] Hombrebueno J. R. , Cairns L. , and Dutton L. R. , et al.Uncoupled Turnover Disrupts Mitochondrial Quality Control in Diabetic Retinopathy, JCI Insight. (2019) 4, no. 23, 10.1172/jci.insight.129760, e129760. 31661466 PMC6962019

[26] Shao Z. , Dou S. , and Zhu J. , et al.The Role of Mitophagy in Ischemic Stroke, Frontiers in Neurology. (2020) 11, 10.3389/fneur.2020.608610, 608610. 33424757 PMC7793663

[27] Silvian L. F. , PINK1/Parkin Pathway Activation for Mitochondrial Quality Control-Which is the Best Molecular Target for Therapy?, Frontiers in Aging Neuroscience. (2022) 14, 10.3389/fnagi.2022.890823, 890823. 35754955 PMC9215347

[28] Zhang J. and Ney P. A. , Role of BNIP3 and NIX in Cell Death, Autophagy, and Mitophagy, Cell Death & Differentiation. (2009) 16, no. 7, 939–946, 10.1038/cdd.2009.16. 19229244 PMC2768230

[29] Pang B. , Dong G. , and Pang T. , et al.Emerging Insights Into the Pathogenesis and Therapeutic Strategies for Vascular Endothelial Injury-Associated Diseases: Focus on Mitochondrial Dysfunction, Angiogenesis. (2024) 27, no. 4, 623–639, 10.1007/s10456-024-09938-4. 39060773 PMC11564294

[30] Zhang X. , Zhou H. , and Chang X. , Involvement of Mitochondrial Dynamics and Mitophagy in Diabetic Endothelial Dysfunction and Cardiac Microvascular Injury, Archives of Toxicology. (2023) 97, no. 12, 3023–3035, 10.1007/s00204-023-03599-w. 37707623

[31] Wu S. , Lu Q. , and Ding Y. , et al.Hyperglycemia-Driven Inhibition of AMP-Activated Protein Kinase α2 Induces Diabetic Cardiomyopathy by Promoting Mitochondria-Associated Endoplasmic Reticulum Membranes in Vivo, Circulation. (2019) 139, no. 16, 1913–1936, 10.1161/CIRCULATIONAHA.118.033552. 30646747 PMC6465113

[32] Wu W. , Zhang X. , and Xing J. , et al.MitoQ Alleviates Prion-Induced Neurodegeneration by Modulating DRP1- and OPA1-Mediated Mitochondrial Dynamics, Free Radical Biology and Medicine. (2025) 238, 582–594, 10.1016/j.freeradbiomed.2025.07.017. 40651617

[33] Wang J. , Zhuang H. , and Yang X. , et al.Exploring the Mechanism of Ferroptosis Induction by Sappanone A in Cancer: Insights Into the Mitochondrial Dysfunction Mediated by NRF2/xCT/GPX4 Axis, International Journal of Biological Sciences. (2024) 20, no. 13, 5145–5161, 10.7150/ijbs.96748. 39430236 PMC11488586

[34] Pu X. , Zhang Q. , and Liu J. , et al.Ginsenoside Rb1 Ameliorates Heart Failure Through DUSP-1-TMBIM-6-Mediated Mitochondrial Quality Control and Gut Flora Interactions, Phytomedicine. (2024) 132, 10.1016/j.phymed.2024.155880, 155880. 39053246

[35] Li Y. , Yu J. , Li R. , Zhou H. , and Chang X. , New Insights Into the Role of Mitochondrial Metabolic Dysregulation and Immune Infiltration in Septic Cardiomyopathy by Integrated Bioinformatics Analysis and Experimental Validation, Cellular & Molecular Biology Letters. (2024) 29, no. 1, 10.1186/s11658-024-00536-2. PMC10826082 38291374

[36] Jiménez-Loygorri J. I. , Villarejo-Zori B. , and Viedma-Poyatos Á. , et al.Mitophagy Curtails Cytosolic mtDNA-Dependent Activation of cGAS/STING Inflammation During Aging, Nature Communications. (2024) 15, no. 1, 10.1038/s41467-024-45044-1. PMC10821893 38280852

[37] Chen S.-Y. , Beretta M. , and Alexopoulos S. J. , et al.Mitochondrial Uncoupler SHC517 Reverses Obesity in Mice Without Affecting Food Intake, Metabolism-Clinical and Experimental. (2021) 117, 10.1016/j.metabol.2021.154724, 154724. 33548253

[38] Gao F. , Zhang Y. , Hou X. , Tao Z. , Ren H. , and Wang G. , Dependence of PINK1 Accumulation on Mitochondrial Redox System, Aging Cell. (2020) 19, no. 9, 10.1111/acel.13211, e13211. 32779864 PMC7511888

[39] Muniyappa R. and Sowers J. R. , Role of Insulin Resistance in Endothelial Dysfunction, Reviews in Endocrine and Metabolic Disorders. (2013) 14, no. 1, 5–12, 10.1007/s11154-012-9229-1. 23306778 PMC3594115

[40] Karar J. and Maity A. , PI3K/AKT/mTOR Pathway in Angiogenesis, Frontiers in Molecular Neuroscience. (2011) 4, 10.3389/fnmol.2011.00051. PMC3228996 22144946

[41] Zorova L. D. , Popkov V. A. , and Plotnikov E. Y. , et al.Mitochondrial Membrane Potential, Analytical Biochemistry. (2018) 552, 50–59, 10.1016/j.ab.2017.07.009. 28711444 PMC5792320

[42] Rethineswaran V. , Jang W. B. , and Choi J. , et al.BAM15, a Mitochondrial Uncoupler Regulates Mitochondrial Function by FUNDC1 Dependently in Hyperglycemia, Research Square. (2023) 1–17, Version 1 (v1) 10.21203/rs.3.rs-2903880/v1.

