# Supplementary figures and images for: FUNDC1‐Associated Regulation of Mitochondrial Function Is Crucial for Preventing Endothelial Injury in Hyperglycemia
Source: Oxid Med Cell Longev. 2026 Jun 10;2026:6619225. doi: 10.1155/omcl/6619225 (PMC13250841; doi:10.1155/omcl/6619225)

Supplementary Figure.1

(a)

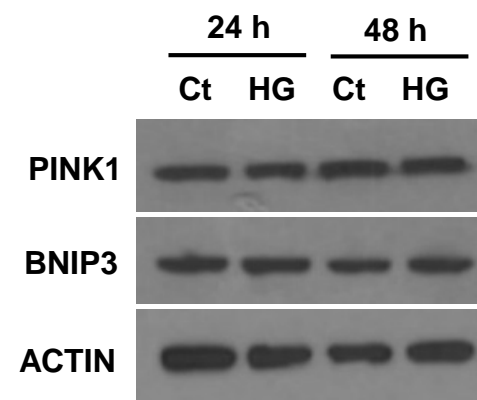

(b)

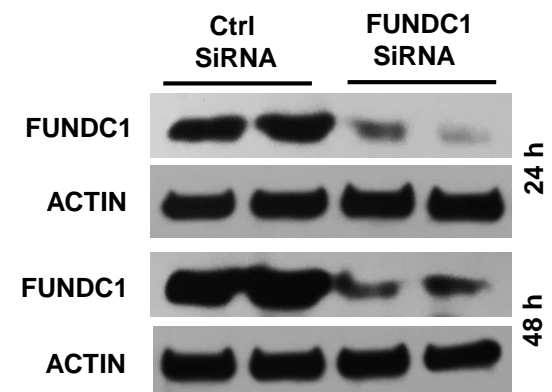



Supplementary Figure.3

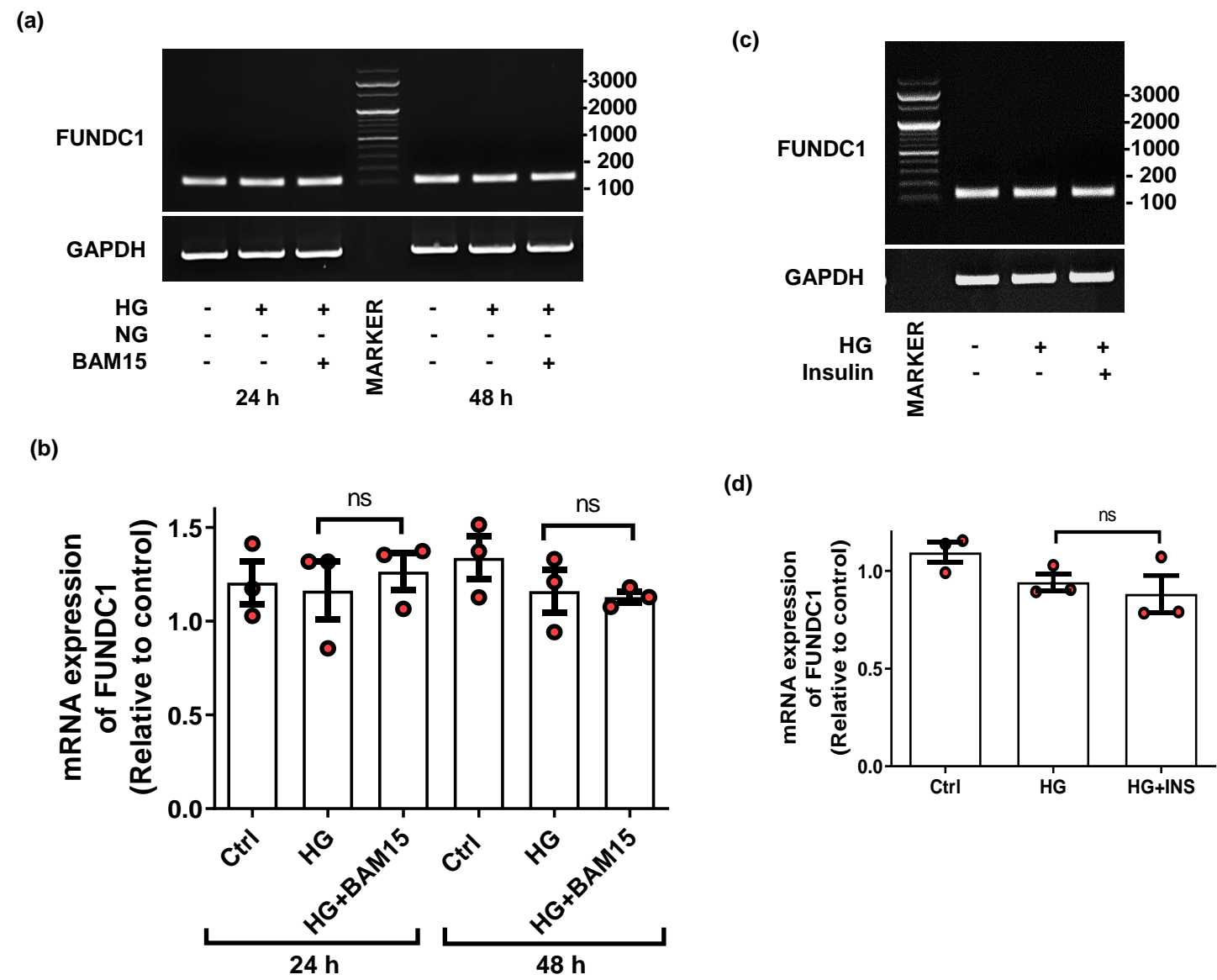

Supplementary Figure.4

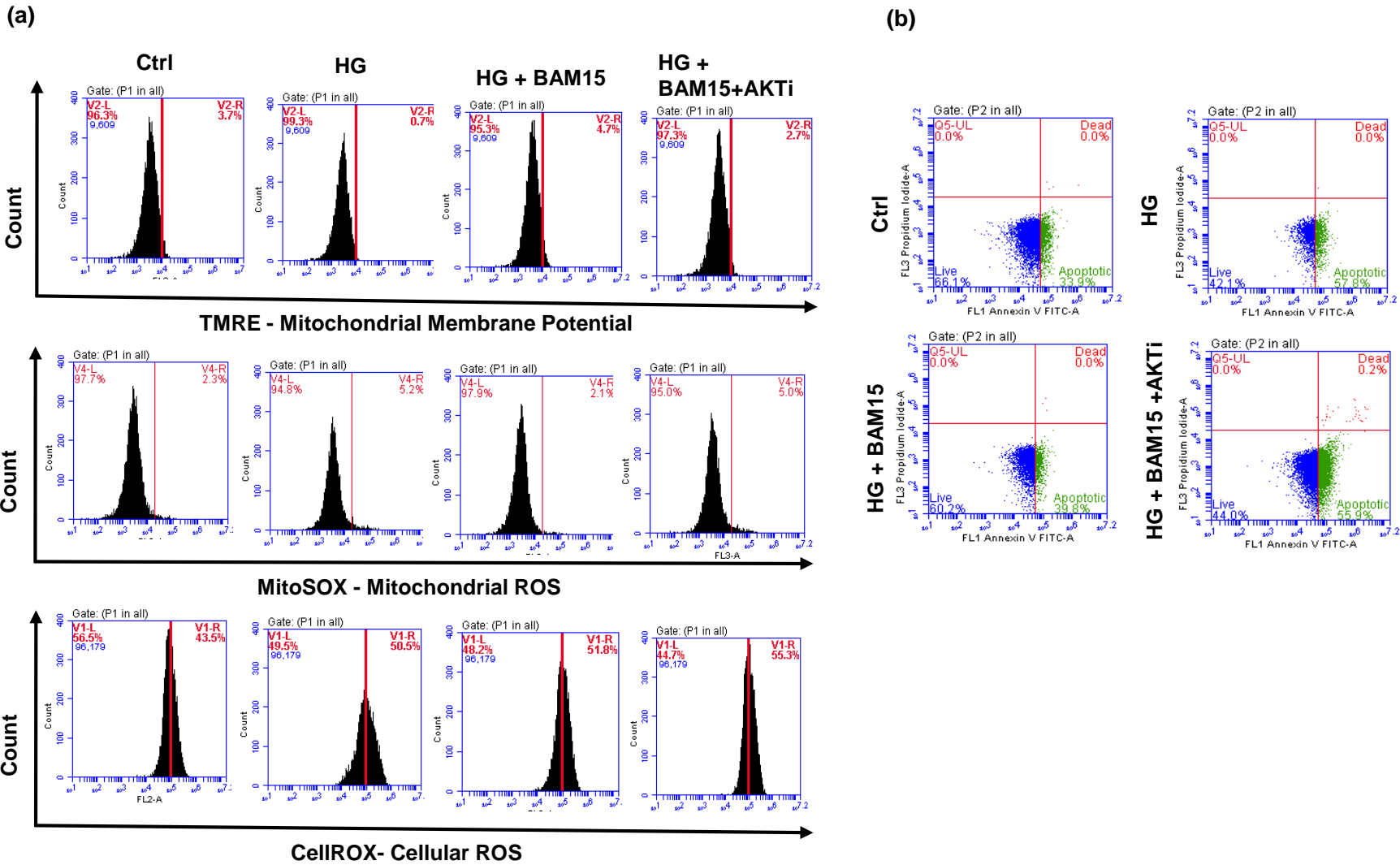

Supplement: Supplementary file 1 — Supporting Information Figure S1: Mitophagy marker expression levels in endothelial cells. (a) Immunoblots of mitophagy markers PINK1, BNIP3, and actin, which were used as loading controls from cells cultured in HG conditions for 24 and 48 h. (b) Proteins were extracted from endothelial cells transfected with siRNA FUNDC1 and control siRNA after 24 and 48 h. The changes in FUNDC1 and actin protein expression levels were visualized using western blotting. The data are presented as the mean ± standard error of the mean (SEM). The results were considered statistically significant at # p < 0.05. p values less than 0.01 or 0.001 are denoted with ## or ###, respectively; NS indicates the difference was not significant. Each experiment was repeated at least three times. Figure S2: FUNDC1 overexpression prevented HG‐induced mitochondrial damage, apoptosis, and cell death. (a) Endothelial cells were transfected with Myc‐tagged FUNDC1, and FUNDC1 overexpression was detected using western blotting with anti‐Myc and FUNDC1 antibodies. (b, c) Cells were transfected with Myc‐tagged FUNDC1, and immunofluorescence was used to observe the expression levels of FUNDC1 Myc with LAMP2. (d–f) Mitochondrial membrane potential and mitochondrial ROS levels were measured using a flow cytometer, TMRE assay, and MitoSOX reagents. (g) The amount of apoptosis and numbers of dead cells were measured using annexin V FITC/PI staining. (h) SOD2 activity was measured in FUNDC1‐overexpressing HG‐treated cells. (i) Data are presented as mean ± standard error of the mean (SEM). The results were considered statistically significant at p < 0.05 (#). p values less than 0.01 or 0.001 are denoted by ## or ###, respectively; NS indicates the difference was not significant. Each experiment was repeated at least three times. Figure S3: FUNDC1 mRNA expression in BAM15‐ and HG‐treated cells. (a, b) Total RNA was isolated and reverse‐transcription PCR was performed to determine the expression levels of FUNDC1 in H [file OMCL-2026-6619225-s001.pdf]
